# Histopathological and Immunohistochemical Study of Neoplastic Cell Heterogeneity in Early and Advanced Ovine Pulmonary Adenocarcinoma

**DOI:** 10.3390/ani15172632

**Published:** 2025-09-08

**Authors:** Raúl A. Reséndiz-Pozos, Jose María González-Saínz, Aurora Ortín, Javier Asin, María Climent, Luis Borderías, Marcelo De las Heras

**Affiliations:** 1 California Animal Health & Food Safety Laboratory, Tulare Branch, School of Veterinary Medicine, University of California-Davis, 18760 Road 112, Tulare, CA 93274, USA; 2 Departamento de Patología Animal, Instituto Agroalimentario de Aragón (IA2), Universidad de Zaragoza, Calle Miguel Servet, 177, 50013 Zaragoza, Spain; jmgsovino@gmail.com (J.M.G.-S.); aortin@unizar.es (A.O.); 3 California Animal Health & Food Safety Laboratory, San Bernardino Branch, School of Veterinary Medicine, University of California-Davis, 105 W. Central Avenue, San Bernardino, CA 92408, USA; jasinros@ucdavis.edu; 4 Departamento de Anatomía, Embriología y Genética, Universidad de Zaragoza, Calle Miguel Servet, 177, 50013 Zaragoza, Spain; mariacli@unizar.es; 5 Servicio de Neumología, Hospital San Jorge, Avenida Martínez Velasco, 36, 22004 Huesca, Spain; lborderiasc@gmail.com

**Keywords:** ovine pulmonary adenocarcinoma, jaagsiekte sheep retrovirus, neoplasm, ovine, lung

## Abstract

Ovine pulmonary adenocarcinoma (OPA) is a naturally occurring lung neoplasia in sheep caused by jaagsiekte sheep retrovirus (JSRV). JSRV infects alveolar type II pneumocytes (ATII) and club cells (CC), and the expression of viral oncoproteins induces a lung adenocarcinoma. The gross pathology of OPA exhibits differences in the anatomical patterns known as classical and atypical forms. Thirty natural OPA tumors, divided equally into early OPA tumors (Group A, GA), atypical tumors (Group B, GB), and classical tumors (Group C, GC), were obtained from adult sheep (2–9 years old). Tumor heterogeneity was studied comparing the histopathology (growth patterns, local invasion, mitotic figures, myxoid nodules), together with immunohistochemistry (IHC) using markers of JSRV-ENV, epithelial cells (ATII cells, CC, ki67), progenitor-stem epithelial cells (K5, p63, CD44), and the anterior grade protein 2 (AGR2). Papillary pattern was predominant in all groups. Lepidic pattern was also relevant in GA, and acinar pattern was relevant in GB. Low proliferation indexes and local invasion were observed in all groups. Myxoid nodules were few. IHC showed that all samples were positive for JSRV-ENV. Cell markers demonstrated that GA was different when compared to GB and GC, showing significantly the highest levels of CC, K5, and p63 positive tumor cells. There were no significant differences between GB and GC. The heterogeneity analysis of OPA tumors revealed that in early tumors, repair is important but is not reflected in classical or atypical different anatomical OPA forms.

## Simple Summary

Ovine pulmonary adenocarcinoma (OPA) is a naturally occurring lung neoplasm of sheep caused by jaagsiekte sheep retrovirus (JSRV). OPA is a relevant disease for the sheep industry and a good translational model because of its similarities with human lung adenocarcinomas. In order to progress in the study of the biology of OPA, we have tried to understand the tumor evolution and the factors influencing the different anatomical forms described in natural cases. We analyzed the histopathology of early and advanced tumors, split into atypical and classical anatomical forms. We also analyzed the heterogeneity of tumor cells using immunohistochemistry. We assessed the number of tumor cells positive for markers for the origin of the neoplasia, progenitor-stem cells, and upregulated proteins relevant to the oncogenic process. We found some differences in the growing pattern, indicating these tumors are infiltrative since initiation, but consistently showing low levels of proliferation. Cell markers of progenitor-stem cells were significantly expressed in early tumors, but there were no differences between atypical and classical anatomical forms. Therefore, tumor cell heterogeneity is not reflected in the anatomical variation. We will continue our search in other elements of the tumors, trying to understand these differences.

## 1. Introduction

Ovine pulmonary adenocarcinoma (OPA; also known as sheep pulmonary adenomatosis and jaagsiekte) is a naturally occurring lung neoplasia in sheep. OPA cases have been recorded in most countries where the sheep industry is important, except in Australia and New Zealand [1,2,3]. The disease has been observed rarely in mouflons and goats [2,3,4]. The tumor cells are derived from alveolar type II pneumocytes (ATII) and/or club cells (CC), secretory epithelial cells of the distal areas of the lung. Two- to four-year-old sheep are primarily affected, and the main clinical signs are oozing of a considerable amount of frothy fluid through the nares, dyspnea, and wasting [2,3,4].

OPA is a contagious disease caused by jaagsiekte sheep retrovirus (JSRV) and can be transmitted experimentally, either using lung fluid, which contains a large number of viral particles or full JSRV molecular clones produced in vitro [2,5]. JSRV is an exogenous simple retrovirus with a genome of 7460 nucleotides containing four basic genes (*gag*, *pro*, *pol*, and *env*), and an additional open reading frame called orfX whose function is unknown [6,7]. JSRV induces transformation through a native envelope structural protein (ENV), which is itself an active oncogene [7].

OPA has been considered a model for human lung cancer because of its similarities with some types of human pulmonary adenocarcinoma, and therefore, it has many translational applications [8,9,10,11]. Furthermore, sheep are considered a good experimental and translational model for many other respiratory diseases [12]. Over the years, significant progress has been made in understanding cell tropism and tissue specificity, cellular receptors, oncogenic mechanisms, diagnostic tests, and the transcriptional response of the lung to JSRV infection [13,14].

Despite these achievements, information about intratumor heterogeneity and its evolution, which aims to expand the understanding of cancer cells, is lacking. Tumors are intricate ecosystems composed of malignant (cancer cells) and nonmalignant (interstitial tissue) compartments, with dynamic and complex interactions between them that determine intratumor heterogeneity [15,16]. The histopathological examination of tumors, in general, and lung tumors in particular, is routinely used as a diagnostic and prognostic tool in both human and veterinary medicine.

The gross findings of naturally occurring OPA are heterogeneous, exhibiting variations in anatomical patterns or forms: classical, atypical, and mixed. Classical forms exhibit extensive, consolidated, grey areas, primarily located in the cranioventral lung regions, and appear moist on section. Atypical forms are characterized by white, well-demarcated nodules that are dry at the cut surface. A wide variety of mixed forms may be observed in the same affected flock, and even nodules resembling atypical lesions have been noted in the same lung in which the classical lesion is present [3,4,17,18]. Histological heterogeneity of OPA may be reflected in the spectrum of the anatomical gross patterns described. Current information about the histopathology of the tumor cells is very scarce, and atypical cases seem essentially the same as classical ones. However, atypical OPA is often more acinar than papillary, and the stroma is more heavily infiltrated by inflammatory cells and connective tissue fibers [4]. In some natural cases of OPA, mesenchymal proliferations of myxomatous tissue have also been described. The myxomatous nodules are usually found together with epithelial cancer cells [4]. They represent a proliferative change associated with viral infection because myxoid cells have positive immunolabeling to the capsid (Ca) or surface proteins (Env) [19,20,21].

Another area of research on natural OPA-related lesions is the investigation of early tumors since very little information is currently available on that topic. In one study, the authors reported that most of the early tumors were white-grey, from 0.8 to 2.5 cm, subpleural, and located with preference in distal areas of middle or caudal lung lobes [21]. The surface of the section showed the same color and was dry, with irregular limits. The tumor line was frequently discontinued due to the presence of smaller satellite nodules, approximately 1 mm in diameter, of the same morphology. In the same study, a different type was described in 1 out of 10 cases and was characterized by intraparenchymatous, sharply demarcated, white nodules about 0.5–0.8 cm in diameter that were bright gelatinous at the cut section. Histopathology of the first type revealed a more complex cancer cell composition, with abundant cells positive to ATII cell markers, a small proportion of cells labeled with CC, and some cells positive to markers of progenitor epithelial cells (i.e., PG6, Keratin 14). Histopathology of the second type of nodule was very similar to that of myxomatous nodules. In another study [22], two types of early lesions were described: a nodular form with 1–2 mm, white, independent nodules similar to the ones described by De las Heras et al. (2014) [21], and the diffuse nodule with small purple-grey areas. Both types were preferentially located subpleurally.

In this study, our objectives were to investigate the OPA tumor histopathology of natural cases, examine the morphology of the neoplasia and neoplastic cell composition, assessed by immunohistochemistry using markers for ATII cells and CC, as well as markers for progenitor cells involved in lung repair. We compared atypical and classical forms, which represent the two ends of OPA lesions. Additionally, we included a group of early OPA. Furthermore, we used the information generated in transcriptional studies in natural and experimental OPA [14], and we employed immunohistochemistry to detect the levels of AGR2 (the most relevant upregulated gene) in tumor cells.

## 2. Materials and Methods

### 2.1. Sample Selection

**Figure 1:**
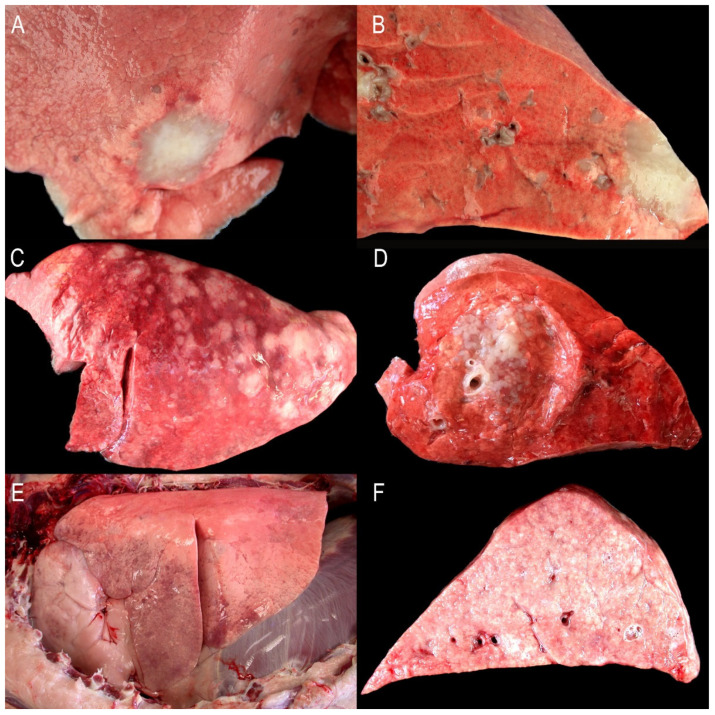
Gross findings of OPA groups: (**A**,**B**) represent Group A with atypical early solitary nodules; (**C**,**D**) represent Group B with atypical extensive forms; (**E**,**F**) illustrate Group C with classical forms.

Naturally occurring OPA cases (*n* = 30) collected between 2010 and 2025 were used in this study. Cases were selected from those submitted for necropsy to the diagnostic service of the University of Zaragoza, Zaragoza, Spain, or obtained from the local abattoir. All cases were adult sheep (from 2 to 9 years old). The cases were classified based on gross criteria as previously described [4,17] and divided into three groups (*n* = 10 each): Group A (GA): Early atypical OPA individual nodules measuring 1–5 cm in diameter; Group B (GB): Atypical extensive OPA, characterized by multiple nodules scattered in one of the lungs; Group C (GC): Classical OPA, found bilaterally and located in cranial and ventral lung areas, with similar extension across all samples (Figure 1). Mixed forms were not included in this study.

Samples of lung from each case were fixed in 10% neutral formalin for 48 to 72 h, embedded in paraffin, sectioned at 4 μm, placed on positively charged slides (Suprefrost Plus, Menzel-Classer, Braunschweig, Germany), and stained with Hematoxylin-Eosin (HE) and immunohistochemical markers (Table 1).

### 2.2. Immunohistochemistry General Procedure

Samples were heated at 60 °C for 30 min before undergoing deparaffinization and hydration according to routine procedures. Samples were then immersed in citrate buffer of pH 6 and heated in a pressure cooker at 121° C for 3 min. After this, sections were allowed to cool down inside the cooker for 1.5 h. Hydrated tissue sections were immersed in Tris-buffered saline (TBS; 0.05 M Tris HCl, 0.15 M NaCl, pH 7.4–7.6) for 10 min three times at room temperature (RT). Peroxidase activity was quenched using Bloxall™ endogenous peroxidase solution (VECTOR Labs, Burlingame, CA, USA) and alkaline phosphatase blocking solution (VECTOR Labs, Burlingame, CA, USA) for 10 min at RT. This step was followed by three additional immersions in TBS for 10 min each at RT. Sections were treated with 2.5% horse serum (VECTOR Labs, No. 30022) for 30 min at RT. After discharging the excess solution, the slides were incubated overnight at 4 °C with the primary antibody diluted in TBS. Normal mouse serum (Mouse IgG, 1–2000 Vector labs) or rabbit serum (IgG Rabbit, 1–100, Vector Labs) at the same dilution in TBS replaced primary antibodies as control for the non-specific reaction. Post-incubation, slides were washed 3 times for 10 min each in TBS (RT) and then covered with ImmPRESS universal polymer peroxidase (VECTOR Labs, No. MP-7500) for 30 min at RT. Following an additional three washes of 10 min each in TBS, the sections were treated with ImmPACT DAB peroxidase substrate (VECTOR Labs, No. SK-4105) and incubated for 3–4 min at RT. Finally, the sections were washed, briefly contrasted with hematoxylin, washed with tap water, dehydrated, and mounted. Normal sheep lymph node or lung tissue sections were used as positive controls, and normal dog lymph nodes were used as negative controls.

### 2.3. Confirmation of JSRV Infection and Investigations of OPA Tumor Histopathology, Growth Patterns, Mitotic Cell Count, Invasion, and Ki67 Labeling

All samples were stained with a mouse antibody against the surface protein of JSRV (JSRV-ENV) [19]. To confirm them as OPA lesions, we categorized the tumor growth into four histological patterns, lepidic, acinar, papillary, and solid, as described in [23]. The median values of these histological patterns were assessed in the 10 cases from each of the three groups. We took 20 random pictures (100× magnification) on two sections from each tumor sample to evaluate the predominant pattern in each field. By combining the data from each case, we obtained the percentage of the histological patterns. Mitotic cell count was performed following international guidelines for mitotic counting in veterinary pathology [24]. Briefly, peripheral areas of the tumor, presumably the most active, were selected. Thus, the mitotic count is indicated for an area of 2.37 mm^2^ (10 high-power fields using a 40× objective and 10× ocular FN 22 mm). The local invasion was evaluated in the 10 cases from each group, identifying individual neoplastic cells or clusters of cells in alveolar spaces near the neoplastic nodule over an area of 2.37 mm^2^. The presence of at least one positive cell or cluster near the neoplastic nodule is enough to consider it positive for invasion.

Four random samples from each group were selected for Ki67 index assessment. The Ki67 index was calculated to indicate the number of positive cancer cells in an area of 2.37 mm^2^. No specific hotspots were found in the tumor samples, and Ki67-positive cells were distributed randomly throughout the tumors. We then counted the number of positive nuclei in cancer cells in 2.37 mm^2^.

### 2.4. Markers for Tumor Cell Assessment

Four cases from each group were selected for this assessment, and tissue sections (4 μm) from each case were processed for immunohistochemistry. They were stained with CD208/dendritic cell–lysosomal associated membrane protein (DC-LAMP) [25] and prosurfactant Protein C (SPC) [26] for ATII; club cell proteins 1 and 2 (CC10-1 and -2) for CC [27,28]; and keratin 5 (K5), tumor protein 63 (p63), and cell surface glycoprotein CD44/Lymphocyte adhesion receptor (CD44) to identify stem cells [29,30] (Table 1).

We took 10 random pictures at 400× magnification on two sections of each tumor sample. In each picture, the number of positive cells and the total number of tumor cells were counted using the ImageJ1.x counting tool. Our data reflect the number of positive cells in relation to the total number of tumor cells. We ensured that more than 1000 tumor cells were counted in each sample.

### 2.5. Anterior Grade Protein 2 (AGR2) Assessment

We conducted a double assessment of AGR2 expression in our samples. Four random samples from each group were selected for this assessment. AGR2 mediates its oncogenic effect through the regulation of other genes, including tumor protein 53 (TP53) and amphiregulin (AREG) [14]. The first assessment involved counting the number of positive and negative cells, following the same procedure used for the other cell markers. Additionally, a semiquantitative estimation was performed using the same method applied in human lung cancer [31]. Briefly, a numerical value was calculated based on staining intensity—not detectable (0), weak (1), moderate (2), and strong (3)—along with the percentage of cells stained at each intensity level (0–100%). A final integrated intensity and frequency value was determined using the formula: 3x + 2y + 1z/100; here, x, y, and z represent the percentage of staining at intensity levels 3, 2, and 1, respectively. Four sections from each group were used in this second assessment.

### 2.6. Statistical Methodology

Data on the histopathology patterns, mitotic count, and Ki67 index of the OPA tumors were presented as frequencies and percentages. No statistical tests were performed on these variables. Markers of secretory cells of terminal bronchioloalveolar respiratory epithelium (markers for ATII [SPC and DC-LAMP] and markers for CC [CC10-1 and CC10-2], markers of undifferentiated/progenitor cells in lung repair (K5 and p63) and markers of upregulated genes with particular interest in OPA tumors (AGR2)) were presented as median and interquartile interval (25–75). Data showed a non-normal distribution even after the refinement of the data and the lack of adequate transformations of the variables. For this reason, analyses were performed by non-parametric tests due to the lack of adjustment with respect to normality. The procedure followed was a first step based on the Kruskal–Wallis test (all groups); when this test was statistically significant, a second step was developed and the differences were analyzed according to the Mann–Whitney U test (group by group). These tests were developed with IBM SPSS Statistics V.29. For all cases, *p* < 0.05 was required to consider statistically significant differences. The data were analyzed using IBM SPSS Statistics (SPSS Statistics for Windows, version 29.0.2.0 (20) (SPSS Inc., Chicago, IL, USA)).

## 3. Results

### 3.1. JSRV-ENV Immunohistochemistry

**Figure 2:**
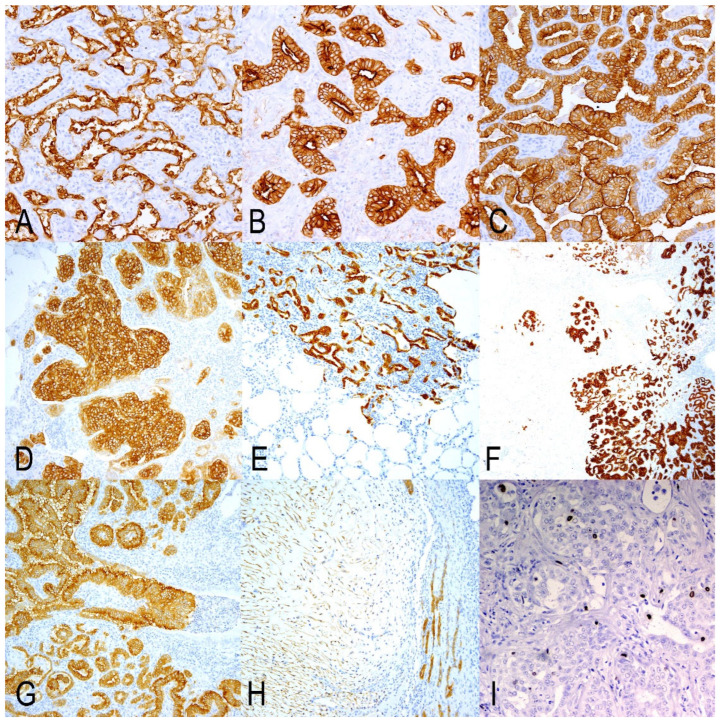
Immunohistochemical analysis of OPA tumors using JSRV-ENV antibody for histopathology and tumor patterns, and Ki67 antibody for proliferative activity. Tumor patterns: (**A**) Lepidic. (**B**) Acinar. (**C**) Papillary. (**D**) Solid. Tumor borders: (**E**) Well-demarcated tumor nodules in Group A. The tumor cells progress into close alveoli in a lepidic way of growing. (**F**) Tumor nodules and cells located in close alveolar areas and separated from the principal tumor nodule. (**G**) Papillary growth inside a bronchiole. (**H**) Myxoid nodule compressing the epithelial component of the OPA tumor. (**I**) Ki67 labeling in tumor cells.

All samples in this study had positive immunolabelling for JSRV-ENV, identifying tumor nodules, small groups of cells, or even single cells located in untransformed alveolar areas or within the bronchioles. The positivity was primarily cytoplasmic but more intense in the cell membranes. The labeling exhibited similar intensity across all growth patterns, though it was particularly intense in the solid tumors. We also discovered positive fusiform cells composing myxomatous nodules (Figure 2H).

### 3.2. Histopathology, Mitotic Count, and Ki67 Index of the OPA Tumors

OPA tumors in GA were primarily subpleural. They were well-demarcated in 2 out of 10 cases; however, in the remaining 8 cases, smaller nodules or clusters of cells were noted in alveolar regions, both close to and distant from the main nodules (Table 2, Figure 2). In this group, tumor cells exhibited a lepidic growth pattern, extending to the adjacent alveoli (Figure 2A). In GB and GC, all cases displayed a similar growth pattern, but with significantly more tumor foci invading or disseminated throughout the normal lung areas, the latter was particularly pronounced in GC. Tumor growth patterns were heterogeneous, comprising combinations of lepidic, acinar, papillary, and solid types, with papillary being the most prevalent among all groups (Table 2). Tumors were primarily located in alveolar areas, although a few bronchioles exhibited JRSV-ENV-positive papillary proliferations, which were more numerous in GC (Figure 2C). Tumor patterns were found either as single-type nodules or as mixed patterns within the same area. The lepidic type represented 32.1% in GA and was sharply reduced in GB (3.3%) and GC (3.9%). The acinar pattern was notable in GB (23.9%), with more limited representation in GA (9.5%) and GC (5.5%). The respiratory epithelium in a variable number of bronchioles displayed papillary patterns and was present in a higher number of bronchioles per section in GC (90.6%). Myxoid nodules were observed in 2 out of 10 samples in GA and in 1 out of 10 in GC (Table 2). The number of mitoses per 2.37 mm^2^ and the Ki67 index were both low, with no clear differences among the groups (Table 1). The distribution of Ki67-positive cells was either random within tumor cells or more numerous in occasional clusters of tumor cells (Figure 2I).

### 3.3. Markers of Secretory Cells of Terminal Bronchioloalveolar Respiratory Epithelium

**Figure 3:**
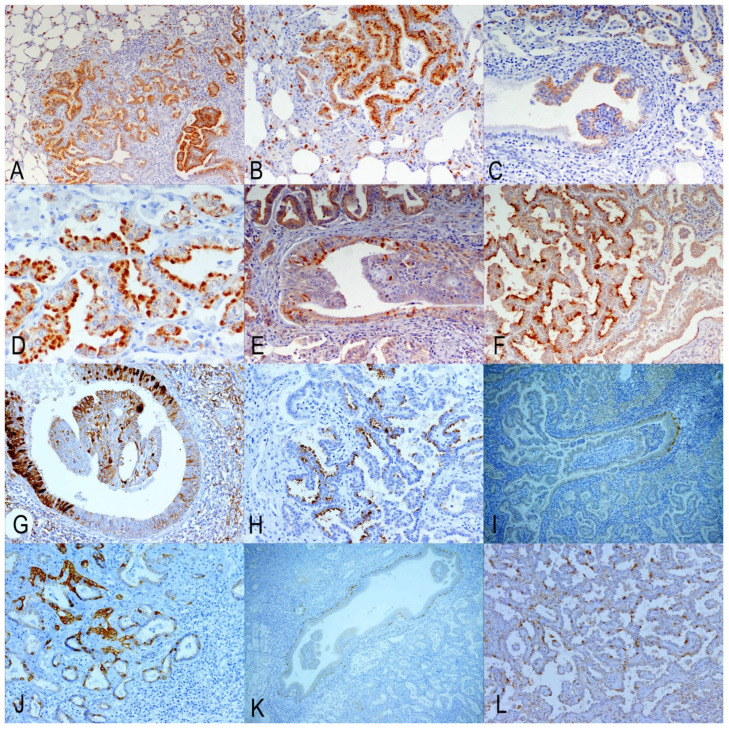
Immunohistochemistry results using markers of secretory cells from the terminal bronchioalveolar respiratory epithelium: (**A**,**B**). ATII markers’ (SPC) positive labeling in alveolar and bronchiolar tumor cells. Positive normal alveolar cells are also shown. (**C**,**D**). ATII marker (DC-LAMP) positive cells are demonstrated in alveolar and bronchial tumors. Normal bronchiolar epithelial cells are negative. (**E**,**F**). Club cell marker 1 (CC10-1) positive cells are located in bronchiolar and alveolar tumors. Groups of negative cells are also present. (**G**,**H**). Club cell marker 2 (CC10-2). Labeled cells can be found in alveolar and bronchiolar tumor cells, together with negative tumor cells. (**I**,**J**). K5 marker demonstrates positive cells in basal untransformed epithelium and groups of tumor cells. (**K**,**L**). p63 labeling in basal cells of bronchiole and in groups of tumor cells.

Markers for ATII (SPC and DC-LAMP) exhibited similar labeling patterns. Consequently, the positivity was granular and cytoplasmic, with greater intensity observed in the apical zone of the cell. Normal alveolar epithelium contained positive cells, while neither bronchiolar nor bronchial respiratory epithelium showed positive cells. The labeling pattern of the tumor cells varied. Positive cells were found in the alveolar and bronchiolar OPA tumors. There was a variable number of cells that were not stained with either marker. Negative cells were either intermixed with labeled cells in certain areas of the tumor or formed completely unlabeled tumor nodules (Figure 3E,F). Solid tumor patterns were consistently negative for both markers. The assessment results revealed significant differences among groups. Both markers indicated that the lowest percentages of positive cells were detected in GA, with increases observed in GB, culminating in the highest percentages in GC. For both markers, these differences were significant when comparing GA to GC. However, differences were not significant between GA and GB, and between GB and GC (Table 3).

Markers for CC (CC10-1 and CC10-2) detected positive cells in the respiratory epithelium of the bronchus and bronchioles, but not in normal alveolar areas. However, CC10-2 exhibited clearer and more intense labeling than CC10-1. Positive cancer cells demonstrated cytoplasmic labeling for both markers. CC10-2 was more concentrated in the apical areas of the alveolar tumor cells. Alveolar and bronchiolar tumors contained positive cells; however, many tumor cells were negative in both locations (Figure 3G,H). Statistical analysis revealed highly significant differences between the groups for the two markers. Both CC10-1 and CC10-2 detected the highest number of labeled cells in GA, with a highly significant reduction in GB and GC (Table 3). CC10-1 did not show significant differences between GB and GC, while CC10-2 demonstrated a highly significant reduction from GB to GC (Table 3).

### 3.4. Markers of Undifferentiated/Progenitor Cells in Lung Repair

K5 antiserum labeled the cytoplasm of basal cells in the epithelial and submucosal glands of normal bronchi. Additionally, positive cells were seldom found in non-transformed bronchiolar and alveolar epithelia. Among cancer cells, positive labeling was observed in isolated instances or in small groups of glandular growths, primarily located in alveolar areas. In bronchiolar tumors, positive cells were present but concentrated in basal regions or scattered among these proliferations (Figure 3I,J). The K5 marker assessment confirmed very small numbers of positive cells in all groups, with the highest number identified in GA. Statistical analysis revealed significant differences among groups; however, GA showed significant differences compared to GB and GC, but significant differences were not found between GB and GC (Table 3).

Antiserum against p63 labeled a complete row of nuclei of basal cells in the epithelium of normal bronchi. This nuclear labeling indicated that cells were also found patchily in the basal cells of the bronchial submucosal glands and the large bronchioles. In neoplastic areas, the distribution was similar to that obtained for K5, but the two markers were not coincident in the same tumor area on many occasions. Positive cells were mainly present in alveolar areas but absent in papilliform structures inside the bronchioles (Figure 3K,L). The p63 marker detected a higher number of cells in GA, which showed statistically significant differences with GB but not with GC. The comparison between GB and GC showed significant differences as well (Table 3).

Using the CD44 marker, only a few interstitial inflammatory cells resembling lymphocytes became labeled, and no tumor cells were labeled.

### 3.5. Upregulated Genes with Particular Interest in OPA Tumors

AGR2-labeled cells were identified in the normal respiratory epithelium of the bronchus and bronchioles. In the bronchus, positive cells were interspersed among mucous secretory cells. Positive cells showed less intensity or were negative in small bronchiolar epithelia. The IHC AGR2 results were similar to those described in other publications [14,31]. In OPA tumors, positive cells were abundant in all samples. Cytoplasmic labeling varied in degree, ranging from high to low, with some tumor gland groups showing no labeling at all. The highest numbers of positive cells were observed in GA, followed by GC. The statistical analysis revealed no significant differences among the three groups (Table 3). The method of semi-quantitative assessment of AGR2 activity in human lung cancer on our OPA samples showed similar values. The indexes for each group were as follows: GA: 1.818; GB: 2.080; and GC: 1.505. According to Alavi et al. (2015) [31], indexes 1 and 2 indicate weak and moderate levels of expression, respectively. Thus, OPA samples showed a moderate level of expression across all groups.

## 4. Discussion

### 4.1. JSRV Infections of Target Cells in Close Lung Areas Are a Key Factor in OPA Progression

The investigation detailed in this paper analyzed the heterogeneity of neoplastic cells of classic and atypical forms of OPA, including early and advanced lesions. All tumors were confirmed to be JRSV-associated via immunohistochemical demonstration of the JSRV envelope protein [19,32]. One remarkable observation from the IHC analysis was that groups of proliferating cells were located in close alveolar areas from the main tumors in GA samples. This demonstrates the infiltrative character of the neoplasia since early development. A very interesting finding was the identification of single JSRV-ENV-positive cells with ATII morphology on the normal alveolar wall or even bronchioles. As the tumor cells are infected with fully replicating JSRV, which can infect and transform adjacent normal ATII, new infections induce new tumors and help in the tumor progression. A proof of the relevance of this mechanism in OPA progression comes from studies on experimental infection of lambs with JSRV-RD (a replicant defective virus), which can infect and induce transformation, but does not replicate, and new infectious particles are not produced. Induced neoplastic nodules had an expansive growth by cellular division with no peripheral invasion [20]. We have confirmed in this study that natural OPA neoplastic cells have low mitotic count and Ki67 index [2,4]. However, OPA lesions seem to progress rapidly. In fact, in a preclinical experimental model of OPA neoplasia, volume doubling times were calculated using computed tomography reconstruction, and the average was 14.8 days [32]. According to these observations, new JSRV infections of targeted cells seem to be the main path of progression for OPA.

### 4.2. Several Histological Changes Can Be Observed in OPA Tumors as Indicators of Tumor Complexity

Several histological patterns indicating low and high potential malignancy coexist in our cases and have been described in other publications [4]. They may represent variations associated with JSRV-ENV protein infection [7,33], as it has been shown that JSRV-RD induces less malignant phenotypes [20]. In some cases, neoplastic cells can metastasize to regional mediastinal lymph nodes and other organs [4,34]. This is an unequivocal indicator of malignancy, reinforcing the idea of the role of other mutagenic phenomena that may also be occurring in OPA tumors. Thus, the discovery of some common integration sites may support the hypothesis of the participation of insertional mutagenesis in the development of the tumor [35]. The influence of each of these factors on OPA progression has not been studied, and we may conclude that the oncogenic potential of the JSRV-ENV could be increased by other changes, potentially leading to metastatic phenotypes.

In human lung cancer (HLC) disease progression, prognosis, and treatment are associated with the appearance of diverse tumor histological patterns. From less malignant to the more undifferentiated, they have been classified into lepidic, acinar, papillary, micropapillary, and solid patterns. The proportion of tumor patterns determines the degree of differentiation, and it is linked to the prognosis of the tumor [36,37]. OPA tumors showed a variety of histologic tumor patterns, but the papillary pattern was the most prevalent in all groups, with some relevance of more undifferentiated patterns in atypical OPA or early tumors. According to the criteria used in HLC, all groups fit into grade 2 [37], with the papilliform pattern being prevalent with less than 20% of more undifferentiated patterns. Several lines of evidence indicate that progression from the lepidic to solid pattern is associated with an increase in markers of cell proliferation and transit to a suppressive immune microenvironment in HLC. Furthermore, non-genetic mechanisms of tumor evolution seem to be determinants of histologic heterogeneity and disease progression [36].

In some OPA cases, myxomatous tissue, presumably of mesenchymal origin, is found associated with epithelial growths, and it is considered part of the OPA tumors [4,19,21]. We assessed the number of myxoid structures that were JSRV-ENV positive in each group, which were present in a few samples. In some cases, myxomatous nodules are detected macroscopically, sharing morphological elements with atypical OPA [21]. However, we have detected them in GA and GC, and they were not detected in GB. Interestingly, GB corresponds with atypical cases, and we may suggest that the morphology of this form is not generally associated with the presence of a mesenchymal component.

### 4.3. Cancer Cell Heterogeneity Does Not Influence the Gross Variations in OPA

A determinant factor of cancer cell heterogeneity is the origin of these neoplastic cells [36,38]. In the case of OPA, two models of experimental infection in lambs have indicated that most infected cells are positive for ATII markers, and in a very low proportion for CC, therefore indicating that these cells are the origin of the OPA [39,40]. We have analyzed the three groups of samples with two specific markers for ATII and another two for CC. Our assessments indicate that in GA, the proportion of CC is significantly higher compared to the other groups. These results correspond with the lowest level of ATII cell markers in this GA. The high proportion of CC positive cells may indicate that they are relevant in the tumor initiation, but their involvement is diluted as the tumor progresses.

A factor that may have influenced our neoplastic cell assessment should be considered. The techniques used may have a limit of detection, and very small amounts of the antigen may have been missed. In order to minimize this effect, we have used two markers specific for the same type of cells, and a similar trend has been recorded in both pairs of markers. Surfactant proteins (SPs) are commonly used to identify ATII cells and are expressed before morphologically distinct ATII cells appear [41]. However, the presence of SPs in these immature cells may also not be detected by IHC, and it remains unclear whether they are part of the negative cell population.

OPA tumor heterogeneity seems to be more complex after our previous immunohistochemical study in early tumors [21]. In this study, together with the number of neoplastic cells positive to ATII and CC markers, a small number resulted positive to p63 and K5, which seem to be markers of cells that participate in the repair of alveolar epithelia [29,42]. In response to injury, specific populations of airway cells localized in the bronchioloalveolar area are mobilized into the alveolar region to participate in repair. These cells are called distal airway stem cells or lineage-negative epithelial progenitors that form p63/K5 to seal the damaged alveoli temporarily. Together with them, bronchioloalveolar stem cells that co-express ATII and CC markers must be shown to reside at the bronchioloalveolar junction and have demonstrated a dual-lineage potential to differentiate into alveolar or bronchiolar cells [42]. ATII cells and CC cells seem to be a complex heterogeneous population involved in either homeostatic maintenance or regeneration after injury [42,43].

In this work, we wanted to assess the proportion of these cells in early tumors and expand the study of the cellular heterogeneity in more advanced OPA tumors. As a result of our assessment, we have found K5 and p63-positive cells in early and in both forms of advanced tumors. Levels of p63 were significantly higher in GA, compared to GB and GC. However, the existence of different isoforms of p63 and the complexity of the role in lung cancer [44] do not allow us to draw any conclusion other than the presence of p63 and k5-positive cells as a part of the heterogeneity of cancer cells in OPA. We also tried to use markers to identify stem cell-like cells in OPA tumors, but IHC is usually insufficient to demonstrate these cells.

In a previous work, we tested putative stem cell markers (CD90 or CD117) by IHC, and tumor cells were negative [45]. This study used CD44, a putative stem cell marker [46]. But no labeling was observed. However, RNA seq analysis indicates that the CD44 gene is upregulated in OPA [14]. In our samples, we identified a few CD44-positive interstitial cells of lymphoid morphology, which may indicate the presence of regulatory T cells [47]. In summary, progenitor cell markers are most numerous in GA, and this fact may relate to how the tumor is initiated, with no clear influence on the generation of classical or atypical OPA.

Information about the transcriptional response of the ovine lung to JSRV infection is reported by Karagianni et al. (2019), and among the cancer genes found upregulated with a particular interest was AGR2, which is a promoter factor of pulmonary and other adenocarcinomas [14]. AGR2 expression is associated with poor prognosis in several cancer types and appears to mediate effects through the regulation of other genes, including tumor pro-tein 53 (TP53) and amphiregulin (AREG), through YAP1, a component of the Hippo pathway [48]. In fact, Hippo pathway activation has been detected in OPA [14]. We have analyzed positive cells in our samples, but none of the assessments showed statistical differences among groups. Therefore, the expression of AGR2 was clear in all groups, but had no influence on the tumor heterogeneity or the anatomical forms analyzed in this study.

As we have mentioned earlier, the relationship between JSRV replication capacity and the characteristics of the JSRV-induced tumors in experimental infections using defective JSRV virus in lambs or mouse models inducing well-demarcated nodular tumors has been described in several papers [20,49]. In contrast, it was also seen that experimentally induced OPA in lambs with JSRV21 molecular clones, with neoplastic foci of different sizes that are adjacent to each other, results in large tumors with a locally invasive appearance [50]. Furthermore, experimental infections in goats develop well-demarcated nodular glandular tumors similar to atypical OPA [51,52]. Thus, well-demarcated tumor nodules, white in color, are described in the lungs of lambs experimentally inoculated with replicating defective virus expressing JSRV-ENV under control of their own LTR produced by transient infection of 293T cells after 4–8 months. Histologically, well-demarcated nodules are seen with glandular proliferation of acinar or papillary pattern [50]. A similar type of tumor can be observed after 5 months of intratracheal inoculation of JSRV21 fully infectious clone in goat kids [50] or kids intratracheally inoculated with concentrated lung fluid obtained from OPA-affected sheep [51,52]. In addition, well-demarcated nodules of papillary adenomas are observed in a mouse model of JSRV infection using a replication-defective adeno-associated virus type 6 that expresses JSRV-ENV protein [19]. However, experimental inoculation with JSRV21 full molecular clone produced by transient infection of 293T cells induced, after 4–9 months post-infection, consolidated grey-purple areas with a gross morphology similar to classical anatomical forms, even in preclinical lesions [32,50].

As we can conclude from these experiments, the reduction or interruption of the replication capacity of the JSRV may be a relevant factor influencing the gross morphology and histopathology of the OPA tumors. However, a few observations in natural cases of OPA showed a similar spectrum of gross morphology described in sheep. Thus, some reports describe similar lesions to atypical or to classical [53]. Naturally JSRV-infected mouflons develop lesions similar to those of sheep [54]. How small ruminant species are infected may influence the development of OPA lesions regardless of the species.

There is a very relevant influence from the heterogeneity of cancer cells and the morphology of the tumors, which is the tumor microenvironment [36,38]. Thus, the next step in this investigation would be the analysis of the OPA tumor microenvironment, which may explain the basis of the variation in the anatomical forms of OPA.

## 5. Conclusions

OPA tumors generally progress rapidly through new infections of targeted cells. Our morphological analysis concluded that the papilliform histological lung adenocarcinoma pattern is the most frequent. However, across all groups, the presence of more malignant patterns indicates the complexity of the oncogenic mechanisms that occur in OPA. We have found that OPA tumors are heterogeneous, with a predominance of cells positive for ATII markers. Early lesions differ when compared to advanced atypical and classical forms, showing the highest levels of CC, K5, and p63-positive cells. This change may indicate that repair is important in the OPA initiation. The heterogeneity of neoplastic cells in OPA tumors does not seem to be reflected in the anatomical forms of OPA.

## Figures and Tables

**Table 1:** List of primary antibodies used in this study.

Antibody	Species	Source	Catalog Number	Dilution
AGR2	Rabbit (P)	Abcam	Ab227584	1:300
CD44	Rabbit (P)	Abcam	Ab24505	1:1000
CC10-1	Rabbit (P)	Biovendor Lab Med Inc	RD181022220	1:2000
CC10-2	Rabbit (P)	Claudio Murgia	Not commercial	1:15,000
DC-LAMP	Rabbit (P)	Immunoytech	PNIM3448	1:2500
JSRV-ENV	Mouse (M)	Dusty Miller	Not commercial	1:500
K5	Rabbit (P)	Biorbyt	Orb-128270	1:8000
Ki67	Rabbit (P)	ThermoFischer Sci.	RM-1906	1:200
SPC	Rabbit (P)	Abcam	Ab28744	1:10,000
p63	Mouse (M)	Novus Biologicals	NB-100-691	1:200

P: Polyclonal; M: Monoclonal; CD44: Cell Surface Glycoprotein CD44/Lymphocyte adhesion receptor; CC10-1: Club cell protein 1.; CC10-2: Club cell protein 2.; DC-LAMP: CD208/dendritic cell–Lysosomal associated membrane protein.; JSRV-ENV: jaagsiekte sheep retrovirus envelope protein; K5: Cytokeratin 5; Ki67: Marker of proliferation Kiel 67, For this marker, we added DAB + NI reagent (SK-4100, Vector Labs) instead of Impact DAB to achieve a black color for better contrast; SPC: Prosurfactant protein C; p63: Tumor Protein 63.

**Table 2:** Histological findings of the tumors distributed by groups. Histopathological tumor patterns in percentages. Local invasion: number of positives/number of cases. Mitosis/2.37 mm^2^ and Ki67 index of proliferation. Number of bronchioles with papilliform tumors. Number of samples with myxoid nodules/total number of samples.

Groups	Histopathology Tumor Patterns (%)				Local Invasion	Bronchial Lesion/Section	Mitoses/2.73 mm^2^	Ki67 Index	Myxoid Nodules
	Lepidic	Acinar	Papillary	Solid					
A	32.1	9.5	51.2	7.2	8/10	1.30	0.088	2.96	2/10
B	3.3	24.0	72.9	0	10/10	0.22	0.166	0.63	0/10
C	3.9	5.5	90.6	0	10/10	2.50	0.077	1.76	1/10

**Table 3:** Cell markers. Significant (bold: highly significant *p* < 0.001; strongly significant: *p* < 0.010, significant: *p* < 0.050), and statistical trend (red) *p* < 0.100.

Marker	Group	Median	Interval (25–75)	Significance (*p*)			
				Groups	GA vs. GB	GA vs. GC	GB vs. GC
SPC	A	0.066	0.0000–0.9571	**0.046**	0.229	**0.017**	0.151
	B	0.318	0.0279–1.0000				
	C	0.852	0.1612–1.0000				
DC LAMP	A	0.009	0.0000–0.9932	**0.030**	**0.071**	**0.019**	0.154
	B	0.386	0.0646–0.9437				
	C	0.869	0.0886–0.9943				
CC10-1	A	0.126	0.0000–0.9931	**<0.001**	**0.003**	**<0.001**	0.251
	B	0.000	0.0000–0.1102				
	C	0.000	0.0000–0.0199				
CC10-2	A	0.087	0.0235–0.7966	**<0.001**	**0.003**	**<0.001**	**0.002**
	B	0.009	0.0000–0.1058				
	C	0.000	0.0000–0.0000				
K5	A	0.000	0.0000–0.0307	**0.004**	**0.030**	**0.003**	0.379
	B	0.000	0.0000–0.0000				
	C	0.000	0.0000–0.0000				
p63	A	0.041	0.0000–0.0239	**0.001**	**<0.001**	**0.088**	**0.024**
	B	0.003	0.0000–0.0088				
	C	0.012	0.0000–0.0646				
AGR2	A	0.850	0.7317–0.9151	0.251	**0.095**	0.322	0.554
	B	0.715	0.4789–0.8958				
	C	0.765	0.5706–0.9267				

## Data Availability

Dataset available on request from the authors.

## Abbreviations

The following abbreviations are used in this manuscript:

OPA	Ovine pulmonary adenocarcinoma
JSRV	Jaagsiekte sheep retrovirus
ATII	Alveolar type II pneumocytes
CC	Club cells
GA	Group A
GB	Group B
GC	Group C
IHC	Immunohistochemistry
AGR2	Anterior grade protein 2
JSRV-ENV	Jaagsiekte sheep retrovirus envelope protein
ENV	Envelope structural protein
Ca	Capsid
RT	Room temperature
HE	Hematoxylin-Eosin
DC-LAMP	CD208/dendritic cell–Lysosomal associated membrane protein
SPC	prosurfactant Protein C
CC10-1	Club cell proteins 1
CC10-2	Club cell proteins 2
K5	keratin 5
p63	tumor protein 63
CD44	cell surface glycoprotein CD44/Lymphocyte adhesion receptor
SPs	Surfactant proteins
HLC	Human lung cancer
